# A rare differential diagnosis of aortic stenosis with a black aortic valve: A case report

**DOI:** 10.1186/s43044-024-00553-8

**Published:** 2024-09-14

**Authors:** Zarin S. Rangwala, Bineesh K. Radhakrishnan, Pruthvi S. Patel, Prasanta K. Dash, G. Gayathri, Vivek V. Pillai

**Affiliations:** 1 Department of Cardiothoracic and Vascular Surgery, Sree Chitra Tirunal Institute for Medical Sciences & Technology, Thiruvananthapuram, Kerala 695011 India; 2 Department of Cardiothoracic and Vascular Anaesthesia, Sree Chitra Tirunal Institute for Medical Sciences & Technology, Thiruvananthapuram, Kerala 695011 India

**Keywords:** Alkaptonuria, Aortic stenosis, Cardiovascular pathology, Ochronosis, Valve replacement

## Abstract

**Background:**

Cardiac ochronosis, presenting as a rare manifestation of alkaptonuria, an autosomal recessive disorder, is characterised by black pigmentation of calcified cardiac valves and atherosclerotic plaques. We report an intraoperative dilemma on the discovery on the black aortic valve in a case of an old lady with degenerative calcific aortic stenosis.

**Case presentation:**

A 60-year-old lady was electively admitted for valve replacement with a bioprosthetic valve for severe aortic stenosis. She was symptomatic with complaints of headache and giddiness and had a pressure gradient of 113/17mmhg across the aortic valve. Intraoperatively, she was found to have cardiac ochronosis on the discovery of pigmented aortic intima extending to the valve leaflets and underwent valve replacement with a mechanical prosthetic valve. She was post-operatively evaluated for the same and diagnosed with alkaptonuria. Though the surgery went uneventful and the patient was discharged without any complication, she was advised to be on regular follow-up to assess valve gradients, paravalvular leaks and to monitor the disease progression.

**Conclusion:**

The presented case sheds light on the rare cardiac manifestation of alkaptonuria. In the absence of definitive pre-operative diagnosis, intraoperative findings played a pivotal role in guiding the surgical approach and choice of prosthetic valve. The decision to use a mechanical valve was influenced by the potential risks associated with bioprosthetic valves in the setting of ochronosis. Ongoing follow-up and monitoring are essential to assess the durability of the chosen prosthetic valve and to manage any long-term consequences of the underlying metabolic condition.

## Introduction

Cardiac ochronosis is a rare manifestation of alkaptonuria, an autosomal recessive disorder caused by a deficiency in homogentisate 1,2-dioxygenase. [1] Initially described by Virchow in 1866, this condition remains of interest due to its rare occurrence and distinctive pigmented tissue deposits. [1, 2] Historically documented only in post-mortem findings, cardiac ochronosis has since been observed in clinical cases and surgical specimens. The condition is typically associated with black pigment deposits in calcified cardiac valves and atherosclerotic plaques. [1] We present a case involving a 60-year-old woman with intraoperative findings suggestive of cardiac ochronosis.

## Case presentation

A 60-year-old woman with a history of hypertension presented with headache and dizziness. Clinical examination revealed a heart rate of 82 beats per minute, blood pressure of 157/70 mmHg, and SpO2 of 100%. She had a body surface area of 1.26 m^2^ and an ejection systolic murmur of grade 4/6 radiating to the carotid arteries. Diagnostic evaluations confirmed severe calcific degenerative aortic stenosis, and she was scheduled for elective aortic valve replacement.

Pre-operative investigations were within normal limits, with a chest radiograph showing clear lung fields and a cardiothoracic ratio of 50%. Electrocardiography revealed sinus rhythm, a heart rate of 90 beats per minute, and left ventricular hypertrophy (LVH). Echocardiography indicated a left ventricular internal diameter of 38 mm in diastole and 20 mm in systole, an ejection fraction of 73%, left atrium size of 33; aorta size of 24 and aortic annulus size of 17 mm. The aortic valve was thickened, calcified, and tricuspid, with a pressure gradient of 113/71 mmHg. Mild mitral and tricuspid regurgitation were present, along with concentric LVH and good biventricular function. The patient preferred a bioprosthetic valve for the replacement.

**Fig. 1:**
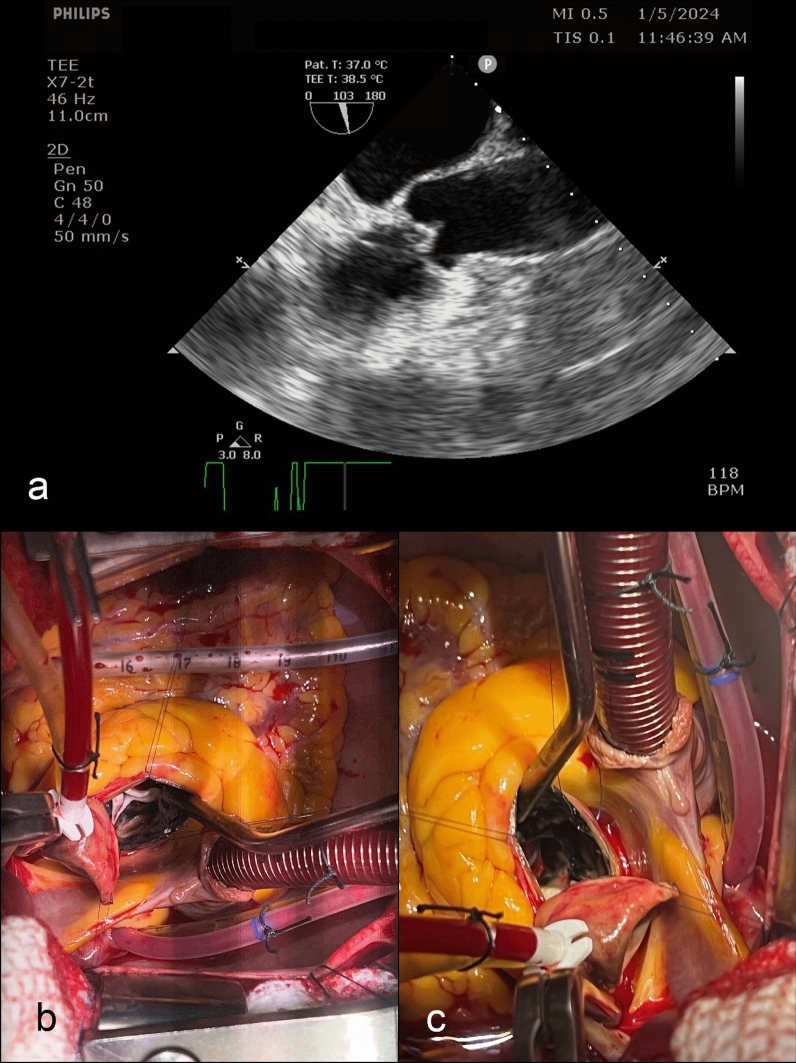
**a** Tranoesophageal echocardiography (TEE) showing no evidence of aortic dissection. Intraoperative findings of the patient. **b** and **c** black pigmentation extending to the aortic valve leaflets and the anterior mitral leaflet (AML) base

Given her small aortic root, the surgical plan included potential root enlargement. During the surgery, we noticed calcified ascending aorta and unusual discolouration over adventitia. Initial suspicion of aortic dissection was ruled out by transesophageal echocardiography (TEE). (Fig. 1a) Cardiopulmonary bypass (CPB) was established using standard aorto-right atrial cannulation, and diastolic arrest was achieved with direct ostial cardioplegia.

**Fig. 2:**
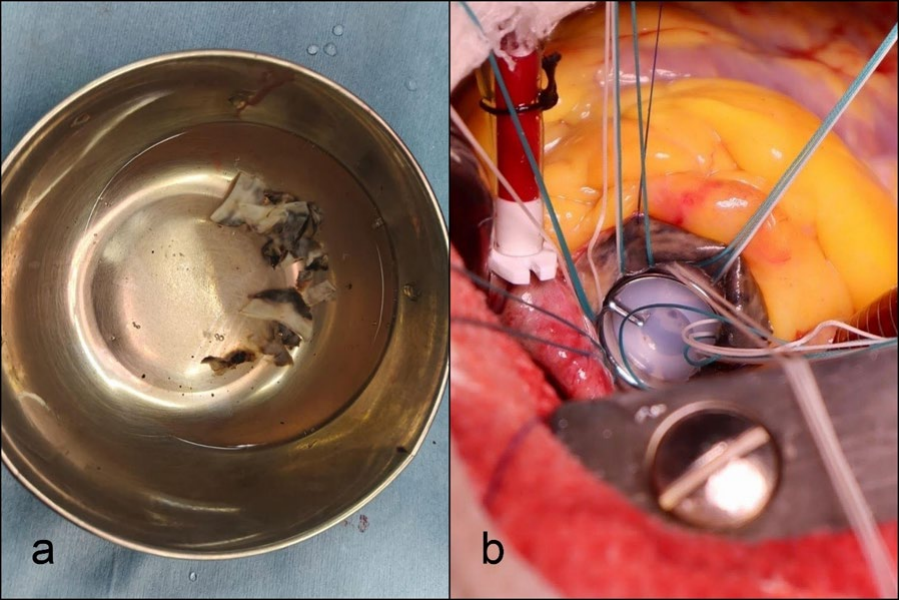
**a** excised aortic valves with black pigmentation; **b** replacement of aortic valve by mechanical prosthetic 19 TTK Chitra aortic valve

The aortic intima exhibited dark green-black pigmentation extending to the coronary origins, aortic valve leaflets, and the base of the anterior mitral leaflet (Fig. 1b, c). The pigmented and calcified aortic valve leaflets were excised (Fig. 2a) and sent for histopathological examination. Although no pre-operative diagnosis of pigmentation disorders or ochronosis was made, the intraoperative findings, combined with the recollection of patient's pigmented sclera, suggested cardiac ochronosis. Urine analysis, which did not produce any discolouration on exposure to room air, revealed black colouration upon addition of 10N sodium hydroxide (NaOH), confirming alkaptonuria. The valve was replaced with a mechanical 19 TTK Chitra aortic valve at supra-annular position (Fig. 2b). Choosing a mechanical valve was a more risk-averse choice since we did not have the confirmed diagnosis and along with that, her having a small aortic root, influenced our decision-making. The decision was made against the use of bioprosthetic valves due to concerns about potential pigmentation effects on bioprosthetic valves and the lack of long-term prognosis data on structural degeneration in such cases.

**Fig. 3:**
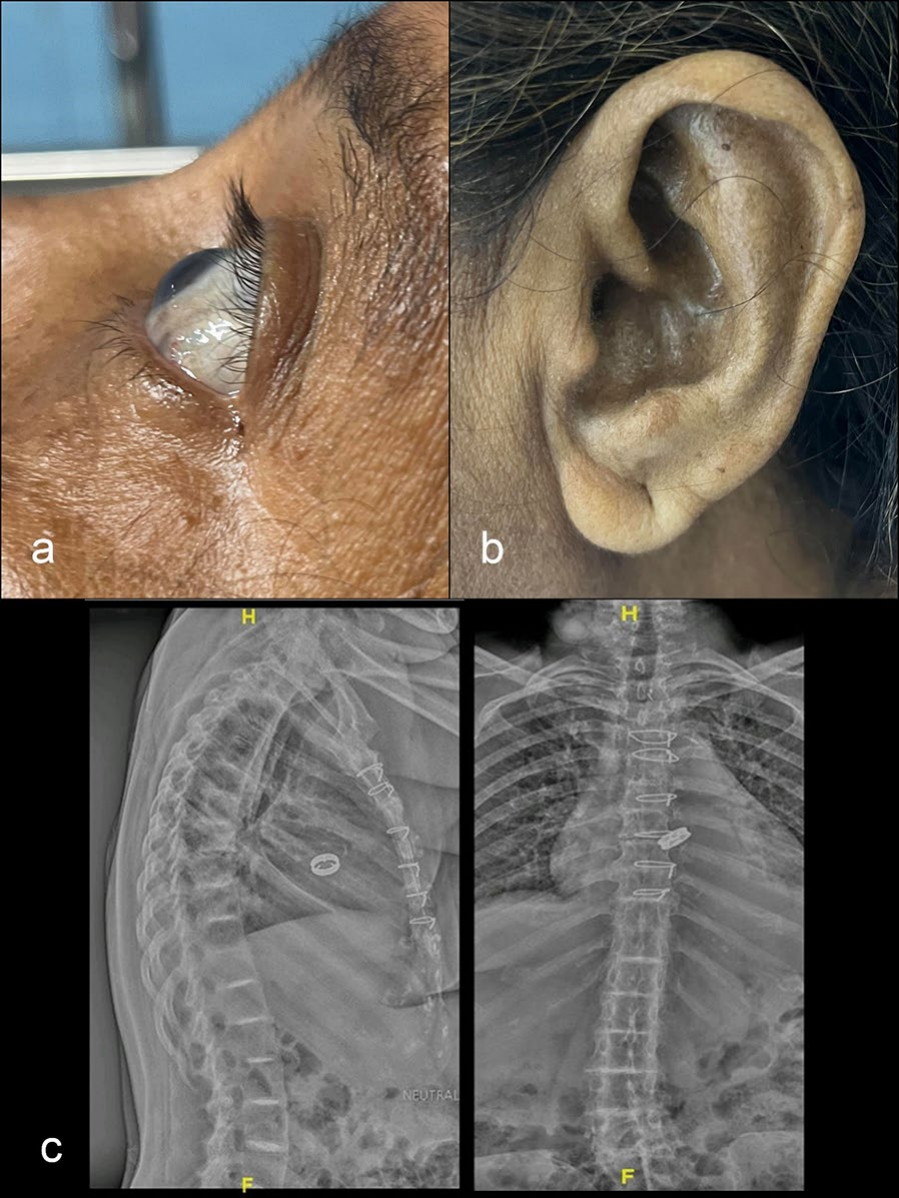
Characteristics of patients. **a** bluish-black discolouration of sclera of eye; **b** bluish-black discolouration of ear pinna; **c** dorsolumbar spine radiograph lateral and AP view showing intervertebral disc space calcifications

Post-operatively, the patient was weaned off bypass with minimal inotropic support and had an uneventful recovery. A retrospective examination revealed bluish-black sclera and ear pinna (Fig. 3a, b). The patient reported a history of black urine in childhood which was never investigated and additionally recent joint pain, which were previously attributed to age-related degeneration. Post-operative spine radiograms showed contiguous dorsolumbar intervertebral disc space calcifications and osteophyte formation suggestive of ochronosis (Fig. 3c). Homogentisic acid detection confirmed alkaptonuria. The patient was discharged on the fifth day with a normal prosthetic valve function, a gradient of 25/16 mmHg, and no paravalvular leaks. At a three- and six-month follow-up, valve gradients remained stable and no paravalvular leaks reported. She was advised to follow a low-protein, vitamin C-rich diet and maintain therapeutic INR levels with regular follow-up to monitor valve function and disease progression.

## Discussion

Alkaptonuria, a rare metabolic disorder with a prevalence of 1 in 100,000 to 250,000, was first described by Sir Archibald Garrod in 1908 and follows autosomal recessive inheritance. [3] Common complications include spondylosis and arthropathies, with cardiac ochronosis being a notable but infrequent complication. [4] Despite over 60 case reports, [1] instances of cardiac ochronosis in Indian literature are sparse, underscoring the importance of accurate diagnosis.

Apart from musculoskeletal involvement, alkaptonuria can present with various clinical manifestations, including ocular and cutaneous pigmentation, genitourinary obstruction due to ochronotic calculi, and cardiovascular involvement [4]. In the cardiovascular system, the calcified aortic valve is one of the most common sites for the deposition of black pigments. This phenomenon is more frequently observed in aortic stenosis than in aortic regurgitation, as calcific deposits are rarer in the latter. Hannoush et al. suggested that deposits of homogentisic acid can increase pressure or turbulence in vessels, damaging microvasculature and leading to dystrophic calcification. [5]

In our case, we observed characteristic black deposits in the aortic intima, including the aortic valve, which were identified intraoperatively. This finding was unexpected as the patient had not been previously diagnosed with alkaptonuria or exhibited symptoms suggestive of the condition. Consequently, the presence of ochronosis posed a dilemma for the predetermined surgical plan of replacing the aortic valve with a bioprosthetic valve. Given the lack of a confirmed diagnosis, differential diagnoses such as aortic dissection, intramural hematoma, or pigment disorders like hemochromatosis are needed to be considered. However, diagnostic modalities such as urine analysis for alkaptonuria and transesophageal echocardiography (TEE) clarified the situation and guided the surgical approach.

Although the patient initially preferred a bioprosthetic valve, we chose to use a mechanical valve due to concerns about potential pigmentation effects on a bioprosthetic valve [6] and the lack of long-term follow-up data on the incidence of paravalvular leaks and structural valve degeneration associated with bioprosthetic valves in cases of cardiac ochronosis. The hazardous effects of ochronosis on cardiac tissue remain unclear, and definitive therapeutic guidelines are yet to be established.

Ather and Roberts reviewed 66 cases of cardiac ochronosis, most of which involved the aorta. [1] While reports commonly document involvement of the aortic valve, there are also accounts of pigmentation affecting the mitral valve, aorta, internal mammary artery, and coronary arteries. Notably, in India, despite the high number of valve replacement surgeries performed, cases of cardiac ochronosis remain exceedingly rare.

Parashi et al. described a case involving alkaptonuric ochronosis of a congenital bicuspid aortic valve in a 38-year-old male patient with diagnosed alkaptonuria who was referred for valve replacement. [7] Their report highlighted the lack of standard guidelines for managing ochronotic valves and selecting prosthetic options. They opted for a mechanical valve due to the patient's age and concerns about the potential degeneration of a bioprosthetic valve caused by ochronosis.

Additionally, black pigmentation of the aortic valve and coronaries was observed in a post-liver transplant patient who underwent coronary bypass grafting and valve replacement with a bioprosthetic valve. In this case, the pigmentation was attributed to medications related to the transplant or changes in dysfunctional liver parenchyma, not alkaptonuria. The rationale for valve selection in this instance was not specified [7].

Reports of bioprosthetic valve replacement in alkaptonuria cases do exist [8, 9], and in those cases, the choice of valve was typically guided by general population guidelines. However, understanding how ochronotic deposits affect valve degeneration and function is crucial for informing future guidelines on prosthetic valve selection for affected patients. The current literature on the accelerated degeneration of biological prostheses and the impact of homogentisic acid (HGA) deposition on their durability is limited.

Although the surgery was uneventful and the patient was discharged without complications, she was advised to have regular follow-ups to assess valve gradients and monitor disease progression.

## Conclusion

The presented case sheds light on the rare cardiac manifestation of alkaptonuria. Our findings underline the critical importance of considering alkaptonuria as a differential diagnosis in patients with unusual pigmentation of cardiac valves and other associated structures, particularly in the context of aortic stenosis. This case exemplifies the need for heightened awareness among clinicians regarding rare metabolic disorders and their potential impact on cardiovascular pathology. In the absence of definitive pre-operative diagnosis, intraoperative findings played a pivotal role in guiding the surgical approach and choice of prosthetic valve. The implications for future research are manifold. There is a clear need for further studies to elucidate the long-term effects of alkaptonuria on both mechanical and bioprosthetic heart valves. The decision to use a mechanical valve was influenced by the potential risks associated with bioprosthetic valves in the setting of ochronosis, highlighting the need for individualised patient management strategies. Ongoing follow-up and monitoring are essential to assess the durability of the chosen prosthetic valve and to manage any long-term consequences of the underlying metabolic condition.

## Data Availability

All the necessary data and material are available in the submitted manuscript.

## Abbreviations

ECG: Electrocardiogram
LVH: Left ventricular hypertrophy
LV: Left ventricle
TEE: Transesophageal echo
CPB: Cardiopulmonary bypass
AML: Anterior mitral leaflet
NaOH: Sodium hydroxide
INR: International normalised ratio
